# Computational fluid dynamic and response surface methodology coupling: A new method for optimization of the duct to be used in ducted wind turbines

**DOI:** 10.1016/j.heliyon.2023.e17057

**Published:** 2023-06-08

**Authors:** Javad Taghinezhad, Shiva Abdoli, Valter Silva, Samira Sheidaei, Reza Alimardani, Esmail Mahmoodi

**Affiliations:** a Department of Biosystem Engineering, Faculty of Engineering & Technology, University of Tehran, Tehran, Iran; b School of Mechanical and Manufacturing Engineering, UNSW, Australia; c Polytechnic Institute of Portalegre, Portalegre, Portugal; d Department of Wood and Paper Science Technology, Faculty of Natural Resources, University of Tehran, Tehran, Iran; e Department of Mechanical Engineering of Biosystems, Shahrood University of Technology, Shahrood, Iran

**Keywords:** Optimization, Ducted wind turbine, RSM, CFD, ANOVA

## Abstract

Wind energy technology, particularly power generation by wind turbines, has received substantial attention due to resource depletion and global warming concerns. These concerns highlight the importance of conducting studies to enhance their efficiency by increasing their power output. The goal of this work was to combine the RSM (Response Surface Methodology (RSM) with CFD (Computational Fluid Dynamics) to discover the optimal design parameters and conditions for ducted wind turbines. To that purpose, twenty-seven runs were chosen using Central Composite Design (CCD) in the design phase. Duct simulation was performed by employing different dimensional parameters and feeding them into a third-order polynomial that fitted to an eight-order function. The analyzed runs discussed the maximum available wind velocity and power at the throat area of the various designed ducts. The wind-enhanced power and speed were studied under different design parameters, and their effects were discussed. The optimum design conditions to capture maximum power were 0.16 m, 2, and 1.5 for design parameters of the duct's throat diameter, contraction ratio, and length-to-throat diameter ratio, respectively. A good selection of design parameters can increase the outpour power up to six times as a general result. By modeling CFD simulations using the RSM method, it is possible to minimize the time and cost of calculation to find the optimized range for the design parameters of the ducts.

## 1 Introduction

Increasing the efficiency of energy production systems that use renewable energy sources is a target for researchers and companies as manufacturers of this equipment. As a kind of renewable source of energy, technology for wind-powered energy is growing very fast because of its promising outcomes in lowering the environmental effect of electricity production. Many studies have analyzed different aspects of power generation through wind turbines, such as moving the energy production site closer to the consumer locations, reducing transmission lines, and changing the energy extraction way from wind energy. The introduction of new forms of harvesting energy from wind has been studied for many years by various researchers.

In this context, ducted wind turbines do not change the original way of energy extraction in conventional turbines. However, they can improve the wind energy capture by enhancing the wind velocity crossing from the throat section of the ducts. The conventional wind turbine may generate more electricity when situated within a duct compared to installation in a free stream. Moreover, the ducts can also reduce the required wind speed to start the wind turbine (as called cut in speed); therefore, the turbine can be in service for a longer time (one year), and total annual energy production increases [1,2].

The research in the wind turbine context has a history of 70 years [3,4]. The main objective of the present study is to enhance wind speed at the installation point of the turbines and reduce the blade's tip losses to boost wind turbines' efficiency [5,6]. Most authors used conical inlets and outlets to increase wind speed. The existing results in the literature show that the cost of power production using ducted wind turbines is half that of traditional wind turbines. Werle and Presz [7] showed that ducted wind turbines could produce power from wind energy higher than the Betz limit. Some researchers have studied the impact of a flanged diffuser and found that adding this feature could increase the flow separation in the duct's wall. This separation reduced the pressure behind the duct and grew air suction into the duct [[8], [9], [10]]. Various optimization and design strategies have been used to boost the output power of ducted wind turbines. For instance, Bontempo has used CFD simulation to analyze ducted wind turbine efficiency [11]. Accordingly, ducted and traditional wind turbines were compared using the actuator disk approach, and the results showed that ducted wind turbines could capture more airflow and wind power. In another study, they worked on installing new types of rotors inside diffuser-augmented wind turbines to analyze their effect on improving performance [12]. Also, the effect of thrust was studied on turbines' efficiency [13] The thrust effect on the duct was found to be essential in increasing extracted power from wind flow.

The two most common analysis methods in this context are Mechanical-Energy-Balance (MEB) and Axial Momentum Theory (AMT) [14]. Some researchers developed an AMT technique and a general model of an actuator disk with a free waring vortextex to analyze the duct-mounted wind turbine characterization. The findings indicated that these turbines could generate more power from the same frontal area with the Betz-defined disk [15]. Another paper studied a computational procedure to analyze the ducted wind turbine performance based on a free-vortex Actuator Disk using a CFD model. They claimed that more than 85% of the stagger angle and the chord of the intake duct may have an impact on the turbine's performance [16]. Additionally, several other researchers used the axisymmetric RANS-BEM approach to optimize the design of shrouded wind turbines [17].

The responsive surface methodology is one of the statistical techniques used to model and optimize different systems in the design and engineering of various equipment [18,19]. The RSM method can give insights into the behavior of system inputs and their relationship to the system's output, known as the response. RSM reduces the required data to model a system [20]. Box and Wilson used RSM [[21], [22], [23]] to produce models with reduced computational costs [24,25]. RSM is an analytical method that models empirical data by investigating the impact of each parameter on response [26]. This study used RSM to achieve optimal performance conditions of CFD output. Using this technique, it is feasible to identify the ideal performance factors or areas where the desired output is shown. This technique minimizes the number of trials necessary to get the best response. It often uses polynomials to illustrate the correlation between the input variables and output [19,27,28]. The first step is to establish an estimation of the function since the relationship between the problem's answers and the components is unclear when using the RSM technique. The estimation begins with the low-order function of parameters. Suppose the linear function of parameters can model the response well. In that case, the first-order function can be considered an approximation function. If there is a curvature in the experimental data, the highest-order polynomial function can approximate the response function. Several works investigate the effect of parameters coefficient in functions. They introduced reduced functions to fit parameters to model response well [29,30].

CFD analyses are suitable for analyzing and optimizing these components and significantly reducing experimental testing costs [31]. The combination of CFD and RSM analyses can reduce the required time to perform calculations and be used as a suitable method for optimization [32]. Rahmannezhad [33] optimized the design of a micromixer utilizing a combination of CFD and RSM. They reported RSM as a practical way to evaluate parameters and their combined effect on the response in the CFD model. Couto [32] used the coupling of CFD and RSM methods to optimize the working conditions of the jet nozzle. Using multi-step optimization, they decreased the number of tests needed to get the desired answer for the design of the jet nozzles. Ansarifard [34] optimized the creation of a radial water turbine to increase efficiency. They optimized the shape of the rotor blade by combining CFD simulation and the RSM optimization technique. They also designed a suitable downstream shape of the turbine to optimize the inlet flow to the rotor. Bourguet [35] introduced an RSM parametric method representing a specific interface between optimization tools and CFD. They optimized the turbine blade profile CFD models using RSM by finding the optimal status for increasing the output power.

In this study, the design of a sample duct for ducted wind turbines was done based on the model introduced by Morel [36] for wind tunnels, and optimized parametric dimensions were found. The duct's parametric dimensions were determined to gain maximum output power in the duct's throat section. In order to achieve the optimal dimensions, the computation time and associated costs were reduced by combining CFD simulations with the RSM statistical estimation approach. The impact of variables such as contraction ratio, throat diameter, and throat length on wind speed and power available in the duct throat (turbine location) was investigated. The best dimensions were found with the least computational cost.

According to the literature review, a few studies include coupled RSM and CFD for various mechanical equipment. It is also found that no work on ducted wind turbines has been done to couple RSM and CFD to solve optimization problems. On the other hand, this approach can be utilized to build a strategy to select the system's design parameters for proper development. As a result, this research aims to fill a knowledge gap by systematically analyzing the influence of duct wall design requirements on the output power of duct-mounted wind turbines using a unique CFD approach coupled with RSM. This goal is achieved via ANSYS CFX, which creates the model and simulates its output behavior.

The goal of the current study is to identify and evaluate how duct length, diameter, and contraction ratio affect the amount of power produced by wind flow in the duct's throat region. The following section presents the study's principles in numerical and empirical methods and defines the data analyzing strategiesThe third section uses CFD and experimental settings to study the effects of the duct shape characteristics on the performance of the device individually. Finally, the investigation of the combined effects of the RSM technique is provided.

## 2 Numerical and experimental setup methodology

### 2.1 Technical background

The inlet section design parameters significantly increase the inlet flow stream to the duct throat section [37]. The inlet section accelerates the wind flow stream. It determines the direction of the inlet flow to the throat section. It can affect the quality of flow delivered into the duct throat part. To minimize the growth of the boundary layers along the wall and reduce the cost of fabricating ducts, the length of the intake part needs to be as short as possible. It must be long enough to prevent flow separation if there is a considerable reverse pressure gradient along the wall [38,39]. We used Morel's method [36,40] for wind tunnel wall design. Then, we matched a third-order to an eight-order polynomial function to model the duct walls.

### 2.2 Modeling of the duct

**Fig. 1:**
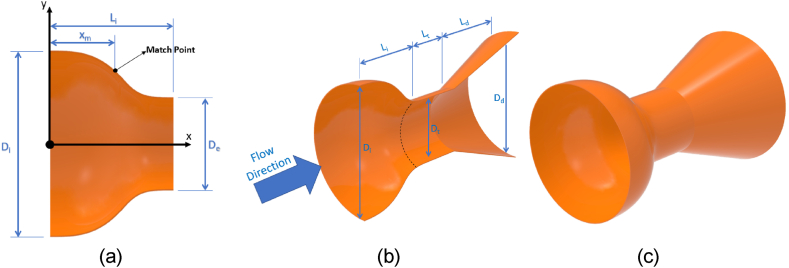
Details for inlet section parameters (a), Main dimensions of the duct (b), 3D view of sample duct.

Fig. 1 shows the required parameters to design the duct inlet section where D_i_ and D_e_ are the duct entrance diameter and the throat section diameters, respectively, L is the entire duration of the shrinkage part, and x_m_ is where the curves' matching point is located (Fig. 1 a and b). The selected range for parameters is as follows; D_e_ chosen values were 0.13, 0.145, and 0.16 m qua blockage effect was less than 20% of the wind tunnel test section. The length of the intake part was.25, 0.75, and 1.5 times D_e_ and shrinkage ratio were picked 2, 3, 4, 6, or 8 when the blockage effect was less than 20% [40]. The design specifications to simulate the ducts are presented in Table 1. All selected ducts were modeled in Catia V5 Fig. 1 (c). After that, CFD calculations for designed models were performed using the Ansys CFX program to examine the flow parameters.

**Table 1:** The designed data to model the ducts.

Model	CR	D_e_	L_i_	Model	CR	D_e_	L_i_	Model	CR	D_e_	L_i_
1	2	16	40	10	4	14.5	36	19	6	13	32.5
2	3	16	40	11	6	14.5	36	20	8	13	32.5
3	4	16	40	12	8	14.5	36	21	10	13	32.5
4	2	16	120	13	4	14.5	109	22	6	13	97.5
5	3	16	120	14	6	14.5	109	23	8	13	97.5
6	4	16	120	15	8	14.5	109	24	10	13	97.5
7	2	16	240	16	4	14.5	220	25	6	13	195
8	3	16	240	17	6	14.5	220	26	8	13	195
9	4	16	240	18	8	14.5	220	27	10	13	195

### 2.3 Mathematical/simulation modeling

**Fig. 2:**
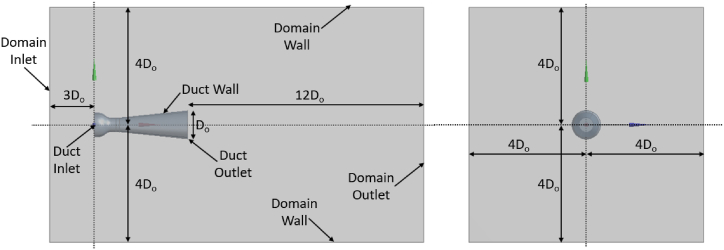
Sketch of optimized numerical domain with its boundary conditions.

A flow simulation model inside the designed ducts was developed using ANSYS CFX software. It was assumed that ambient and wind flow temperature was constant at 300 K. At the system's inlet section, the air has a density of 1.225 kg/m^3^ and an absolute pressure of 1 atm, making it a non-compressible Newtonian flow. Regardless of the size of the domain, the solution to the problem may be found by using the optimized simulation domain shown in Fig. 2. The domain characteristics are considered as a cube, such as values were used by Giahi and Pinto [41,42].

The Shear Stress Turbulence (SST) method was utilized to anticipate the aerodynamics and specifications of flow through the duct. The SST approach can precisely analyze the boundary layer due to its high capability to research the impacts of several elements on fluid flow, such as pressure fluctuations and flow turbulence. For numerical modeling, RANS equations were utilized, which coupled Navier-Stokes and continuity functions with the finite volume technique model. Eq. (1) shows the fluid flow continuity equation. The RANS governing equation comprises the Navier-Stokes and the continuity functions, which are able to be expressed as mean quantities (Eq. (2)).

1 $$\frac{\partial \rho u_{j}}{{\partial x}_{j}}=0$$

2 $$u_{j}\frac{{\partial u}_{i}}{{\partial x}_{j}}=-\frac{1}{\rho }\frac{\partial p}{{\partial x}_{j}}+\frac{\mu }{\rho }\frac{\partial^{2}u_{i}}{{\partial x}_{j}{\partial x}_{j}}-\frac{\partial }{{\partial x}_{j}}\left(\bar{u_{i}^{\prime }u_{j}^{\prime }}\right)$$

The SST turbulence model used in this study is presented by following two transport equations (Eq. 3 and Eq. (4)):

3 $$\frac{\partial \left(\rho k\right)}{\partial t}+\frac{\partial \left(\rho u_{j}k\right)}{{\partial x}_{j}}=\frac{\partial }{{\partial x}_{j}}\left(\Gamma_{k}\frac{\partial k}{{\partial x}_{j}}\right)+G_{k}-Y_{k}+S_{k}$$

4 $$\frac{\partial \left(\rho \omega \right)}{\partial t}+\frac{\partial \left(\rho u_{j}\omega \right)}{{\partial x}_{j}}=\frac{\partial }{{\partial x}_{j}}\left(\Gamma_{\omega }\frac{\partial \omega }{{\partial x}_{j}}\right)+G_{\omega }-Y_{\omega }+D_{\omega }+S_{\omega }$$

where G_k_ denotes the creation of kinetic energy of chaotic flow related to average speed fluctuations, and $G_{\omega }$ denotes the creation of ω , as defined below (Eq. (5) and Eq. (6)):

5 $$G_{k}=-\rho \bar{u_{i}^{\prime }u_{j}^{\prime }}\frac{\partial u_{j}}{{\partial x}_{i}}$$

6 $$G_{\omega }=\alpha \frac{\omega }{k}G_{k}$$

$\Gamma_{k}$ and $\Gamma_{\omega }$ calculate the efficient dispersibility of *k* and ω, and represent as follow (Eq. (7) and Eq. (8)):

7 $$\Gamma_{k}=\mu +\frac{\mu_{t}}{\sigma_{k}}$$

8 $$\Gamma_{\omega }=\mu +\frac{\mu_{t}}{\sigma_{\omega }}$$

where σ_k_, $\sigma_{\omega }$, and μ_t_, the Prandtl values for k, ω, and the viscosity of turbulent flow, are calculated as below (Eq. (9)):

9 $$\mu_{t}=\frac{\rho k}{\omega }\frac{1}{\max\left[\frac{1}{\alpha^{*}},\frac{\Omega F_{2}}{a_{1}\omega }\right]}$$

Y_k_ and $Y_{\omega }$, the dispersion coefficients for k and ω, are computed as below (Eq. (10) and Eq. (11)):

10 $$Y_{k}=\rho \beta^{*}f_{\beta *}k\omega$$

11 $$Y_{\omega }=\rho \beta f_{\beta }\omega^{2}$$

$D_{\omega }$ is Cross-Diffusion modification and is presented by (Eq. (12)):

12 $$D_{\omega }=2\left(1-F_{1}\right)\rho \sigma_{\omega ,2}\frac{1}{\omega }\frac{\partial k}{{\partial x}_{j}}\frac{\partial \omega }{{\partial x}_{j}}$$

finally, S_k_ and $S_{\omega }$ are user-defined terms. Calculating these equations makes it possible to complete the SST turbulence model.

The upwind technique solution was developed to control and reduce errors and faults in numerical calculation. This domain considers the walls, outlet, inlet, and interface boundary conditions. The boundary condition in the walls was regarded as a No-Slip wall. Also, it should be stated that due to lower wind speed in this research, the flow is always Subsonic. The computational domain's intake wind flow pattern was considered to have a 10 m/s velocity and a 5% intensity in the free stream flow range. The duct inlet and outlet sections were connected to open stream flow using the General Grid Interface (GGI), and the walls were set to the No-Slip wall. Conservative Interface Flux (CIF) was determined for the turbulent flow at the duct's intake and output in comparison with freestream flow [43]. At the domain's flow outcome, the pressure boundary conditions were taken into consideration. At the boundary layers, the pressure was locally measured using the static average pressure. At the last stage of the domain setting, the residual goal for the convergence criterion of the simulated duct was set at 10^−4^. With this regard, each wind turbine had a constant resistance to the flow entrance to the duct; to reduce calculation cost and time, we did not model the wind turbine in the duct, as many other researchers omitted the turbine from the duct [[44], [45], [46], [47]].

#### 2.3.1 Mesh independency

**Fig. 3:**
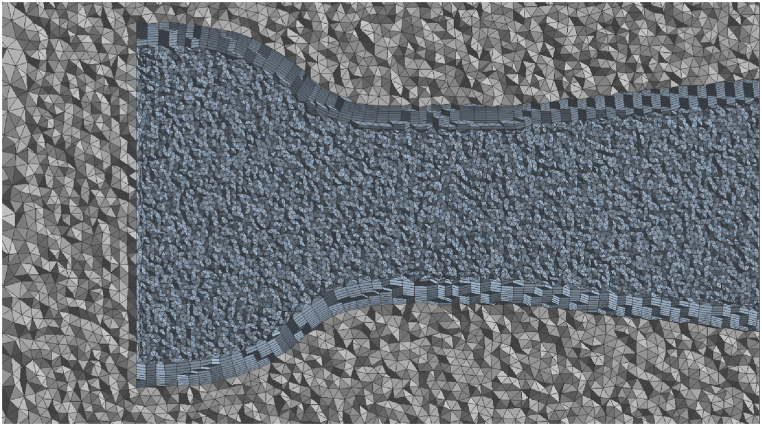
A part of the grid layout and details.

The ANSYS meshing software was used to build a non-uniform mesh for the ducts and their domain [28]. The discrete domain was optimized for the number and form of cells, and the final mesh is shown in Fig. 3. In areas near the wall, the study of flow stream velocity and pressure changes due to boundary conditions requires elements with a higher resolution ratio [32], observed in the computational domain mesh as a fine mesh.

Examining mesh independence is one of the essential parts of any simulation in engineering analysis software such as Ansys. All modeling programs that split the model domain into smaller sub-domains should consider the solver's independence from the domain's mesh. If the assessment of mesh independence is not performed, the simulation results are unreliable [32]. Mesh independence refers to choosing the optimal mesh for the correct answers. In other words, if the mesh is too coarse, the simulation results will almost be unreliable. If the mesh is too fine, the correct solutions will probably not be converged because of the rounding errors and high cost of computational systems.

**Fig. 4:**
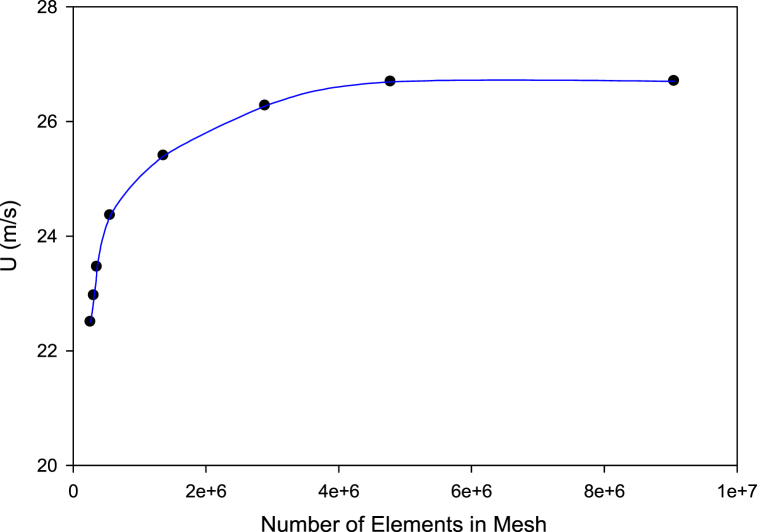
Convergence of wind velocity at the throat (the average over the area) of the sample duct at various numbers of elements in the mesh.

To explore solution independence from the mesh grid, variables such as temperature, speed, and pressure, should be selected according to the problem and studied in different mesh sizes. In this study, eight variations of the mesh size were evaluated, as illustrated in Fig. 4. The wind velocity predicted at the throat section of several ducts in the various numbers of mesh elements at the computational domain of the duct is shown in this figure. The calculated wind speed converges to extend the mesh pieces, and the difference between the calculated wind speeds is minimal. Therefore, from the number of elements above 5 million, the obtained response is independent of mesh. During mesh sizing Y+ was arranged to be lower than 1 with the growth rate of 1.1 in all models.

### 2.4 Design of experiment

This research used Central Composite Design (CCD) of RSM to determine the response surface. CCD with n parameters (X_1_, X_2_, …, X_n_) included three sections: a factorial design included 2^n^ points, the axial section included 2n points, and the number of runs repeated at center points [48]. These sections shall be specified to make a CCD. The following equation (Eq. 13) shows a second-order (quadratic) model for n parameters:

13 $$Y=\beta_{0}+\sum_{i=1}^{n}\beta_{i}X_{i}+\sum_{i=1}^{n}\beta_{ii}X_{i}^{2}+\sum_{i=1}^{n}\sum_{j=i+1}^{n}\beta_{ij}X_{i}X_{j}+\varepsilon$$

β_0_ represents the constant of the equation, β_i_ represents the linear constant multiplier, β_ii_ represents the quadratic constant multiplier, β_ij_ represents the interactive constant multiplier, and ε represents the experimental error. RSM was analyzed using Design Expert 12.0 operating software.

### 2.5 Data analysis

The software package of Design Expert 12.0 was employed in this study's analysis of empirically gathered data and approximations of model component coefficients. The value of the coefficients of the specified model components was estimated using a one-way ANOVA (analysis of variance). Additionally, depending on the suggested model variables, ANOVA is utilized to assess the sufficiency and fitting of results to input parameters. A model with significant results has a *P*-Value lower than 0.05 [49]. The suggested model's validity is measured by the coefficient of regression (R^2^), and its degree of statistical significance is evaluated by the F value. The value of pure errors and residuals at repeated points was investigated using the lack-of-fit test. As a validation parameter, the estimated residual sum of squares was utilized to make sure the developed model matched the experimental data. Equations and data processing used in this investigation were identical to those used by Prakash Maran [48] and Pashaei [50].

## 3 Result and discussion

### 3.1 CFD analysis

**Fig. 5:**
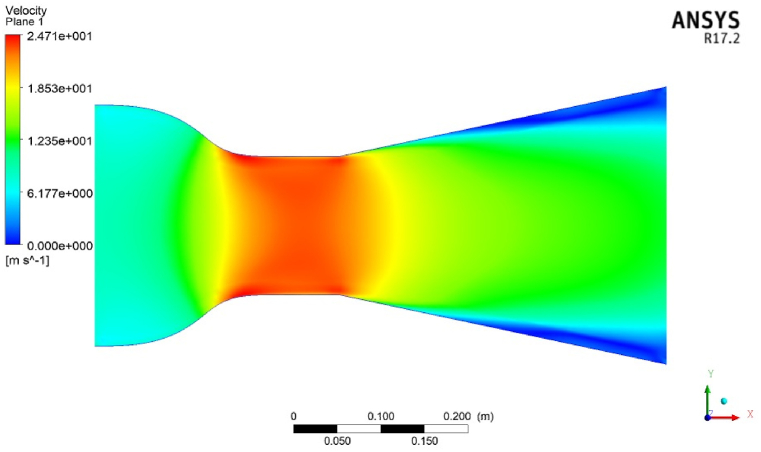
The contour of velocity for a new duct.

The effects of design parameters for one of the designed ducts on the velocity contour are presented in Fig. 5. From this plot, it is shown in the proposed duct that the wind flow velocity accelerated continually, increasing to more than two times of inlet wind speed. A flowing stream with constant contraction and expansion can enhance wind velocity at the throat section. The various models simulated in the experiment were based on the parameters of duct throat diameter, length-to-throat diameter ratio, and contraction ratio. They adjusted to predetermined values, and the input wind speed factor was constant (10 m/s) for all designed ducts. The result of the CFD calculation was analyzed by the RSM method. Validation of CFD analysis by doing experimental tests for the designed duct was done by Refs. [49,51,52] previously.

### 3.2 ANOVA analysis

To examine the effect of designing elements on duct efficiency, a model was created by using ANOVA analysis. ANOVA is a collection of statistical tools investigating the mean in clusters and their related functions (For example, variation within or between groups). F-test was applied to calculated data to check and confirm the adaptation of the reduced quadratic model on two factors, the wind speed and power output at the throat section. The ANOVA for modeling enhanced wind velocity obtained at the duct's throat is provided in Table 2. The created model is significant with an interval of confidence of 95% if the p-value is less than 0.05. It indicates that the current prediction is statistically significant, indicating that the design elements have a direct influence on the airflow speed in the duct's throat region. The coded form for A, B, and C variables are shown as throat section diameter (D_t_), Contraction ratio (A_r_), and length ratio (L_r_), respectively. A_r_ is the proportion of the duct's output and entrance area, and L_r_ represents the ratio of the duct length to the throat diameter. Model significance and proper variable fitting are confirmed by the model's F-value of 175.96. There is barely a 0.01% probability that an F-value with this great value may arise because of noise.

**Table 2:** ANOVA table for enhanced wind speed model.

Source	Sum of Squares	df	Mean Square	F value	p value	
**Model**	218.18	9	24.24	175.96	<0.0001	significant
A-D_t_	2.35	1	2.35	17.05	0.0020	
B–A_r_	2.64	1	2.64	19.15	0.0014	
C–L_r_	130.90	1	130.90	950.10	<0.0001	
AB	0.1315	1	0.1315	0.9547	0.3516	
AC	0.8228	1	0.8228	5.97	0.0346	
BC	0.5793	1	0.5793	4.21	0.0674	
A^2^	1.18	1	1.18	8.56	0.0152	
B^2^	0.1290	1	0.1290	0.9363	0.3561	
C^2^	0.0554	1	0.0554	0.4019	0.5403	
**Residual**	1.38	10	0.1378			
Lack of Fit	1.38	5	0.2755			
Pure Error	0.0000	5	0.0000			
**Cor Total**	219.56	19				
Std. Dev.	0.3712		R^2^	0.9937		
Mean	13.05		Adjusted R^2^	0.9881		
C·V. %	2.85		Predicted R^2^	0.9248		
			Adeq Precision	49.9571		

R-squared, an estimate of the variance value around the mean shown by the simulated model for enhanced wind velocity, is provided in Table 2. The values near 1 indicate that the model matches the data effectively. Table 2 shows that the R2 value is more than 0.99, indicating that the statistical model addresses the data with excellent precision. The Predicted R^2^ of 0.9248 and the Adjusted R^2^ of 0.9881 are reasonably in agreement, meaning that the deviation is less than 0.2. To assess if the accuracy is sufficient, the range of the predicted values at the design points is contrasted with the average prediction error. Reported value ratios higher than 4, 49.96, for this model demonstrate appropriate model classification [50].

The same analysis was carried out to demonstrate how design factors affected the power. The power was available at the duct's throat and is calculated based on a similar calculation in Ref. [53]. The effect of design factors on the available power is shown in Table 3. The model's reported F-value of 203.59 indicates that the variables can correctly predict the response. The discrepancy between the Predicted and the Adjusted R^2^ is less than 0.2, which is considered a fair agreement. The adequate precision, or signal-to-noise ratio, is 49.7802; anything above 4 denotes excellent reliability. Then, the presented model helps exploring the design parameters to find an optimized condition.

**Table 3:** ANOVA table for the calculated available power model.

Source	Sum of Squares	df	Mean Square	F value	p value	
**Model**	21504.63	9	2389.40	203.59	<0.0001	significant
A-D_t_	1158.90	1	1158.90	98.74	<0.0001	
B–A_r_	27.57	1	27.57	2.35	0.1564	
C–L_r_	387.91	1	387.91	33.05	0.0002	
AB	15.08	1	15.08	1.29	0.2834	
AC	956.22	1	956.22	81.47	<0.0001	
BC	5.56	1	5.56	0.4737	0.5069	
A^2^	380.69	1	380.69	32.44	0.0002	
C^2^	142.90	1	142.90	12.18	0.0058	
A^2^C	517.30	1	517.30	44.08	<0.0001	
**Residual**	117.37	10	11.74			
Lack of Fit	117.37	5	23.47			
Pure Error	0.0000	5	0.0000			
**Cor Total**	21621.99	19				
Std. Dev.	3.43		**R**^**2**^	0.9946		
Mean	30.41		**Adjusted R**^**2**^	0.9894		
C·V. %	11.27		**Predicted R**^**2**^	0.8991		
			Adeq Precision	49.7802		

The following are the completed equations (Eq. (14) and Eq. (15)) for the average wind speed in the duct throat and the available power:

(14) $$U=12.851+1.242*A-1.558*B+3.756*C+1.13*AB+0.594*AC-0.671*BC+1.288*A^{2}+0.884*B^{2}-0.141*C^{2}$$

(15) $$P=20.615+23.876*A-4.619*B+13.817*C+4.034*AB+20.359*AC-2.099*BC+17.718*A^{2}+7.03*C^{2}+17.983*A^{2}C$$

**Fig. 6:**
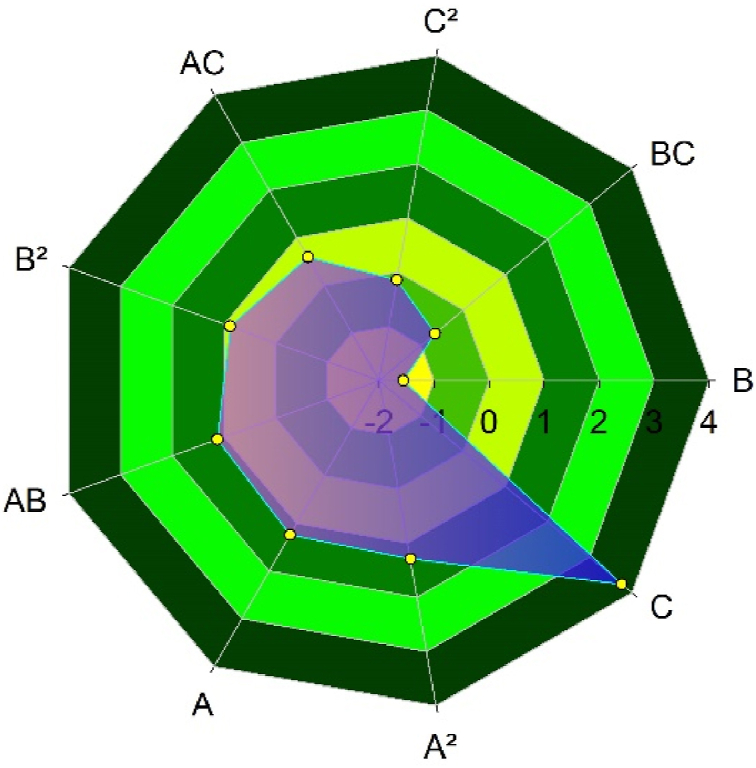
Spider web chart for design parameters and interactions and their effects on the mean velocity of wind (m/s) inside the duct throat.

In these equations U is the mean speed of the wind (in m/s) at the duct turbine section, P represents the power that is available in the same place, A represents the diameter of the throat section (in m), B is the reduction proportion (the ratio of the area of the entrance air to the area of the throat section), and C represents a duct's length proportion (the ratio of the length of the entire duct to the diameter of the throat). ANOVA results and spider web chart indicate that parameter C (Duct Length Ratio) significantly influences average wind velocity (Fig. 6). Further parameters and interactions are significant, but in comparison with parameter C, their effect is low. It indicates that the development and rise of the intake wind velocity to the throat are significantly impacted by the inlet section's entire length in relation to the throat diameter. The stream flow must have sufficient space to develop before entering the throat.

Parameter B (the contraction ratio) has a negative influence on the computed output; also, its interaction with parameter C (BC) has a negative influence on the output. Parameter A (duct throat diameter) positively affects response when its quadratic affects it. In contrast, its interaction with B and C (AB and AC) has lower positive effects on response. The quadratic of the C parameter has a positive influence on the response. However, its impact is the smallest between positive coefficients.

**Fig. 7:**
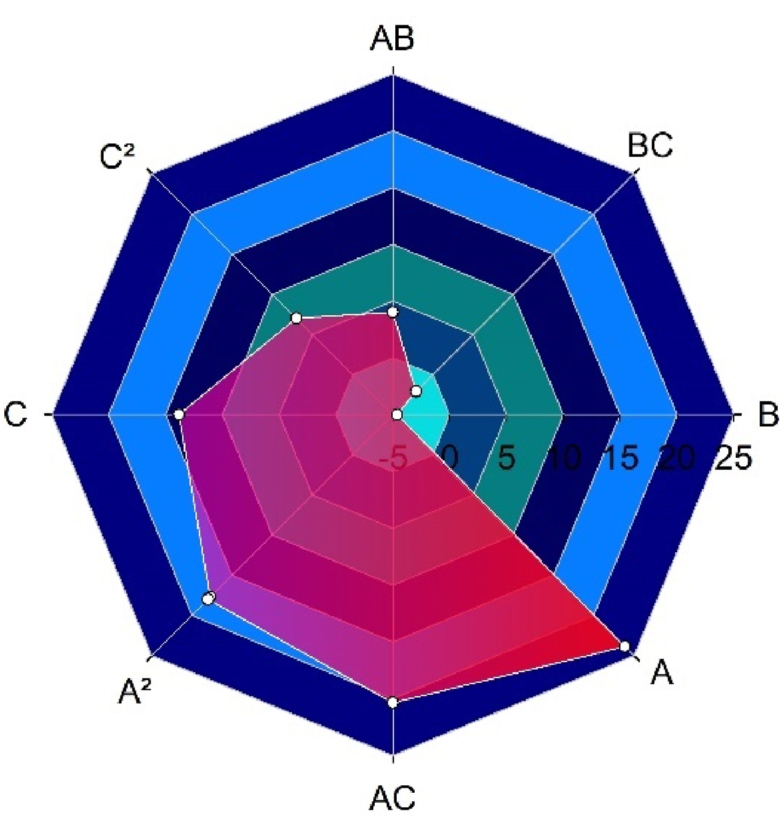
Spider web chart for design parameters and interactions and their effects on average available power inside the duct throat.

ANOVA results and spider web chart indicate that parameter A (duct throat diameter) positively affects available power, and its effect is prominent (Fig. 7). Its combination with C (Length Ratio) positively affects the response. Its quarter is in the following position of influential factors: the impact of the throat area on power response. The positive effect of parameters C and C^2^ are shown in the impact of duct length and its quarter on response. It means that the power can be extended with an increase in length ratio compared with the low duct length. Parameter B (the contraction ratio) negatively affects the response. In contrast, its interaction with parameter C reduced the adverse effect. It means the negative effect of the contraction ratio is more effective than the positive effect of the duct length ratio. Parameter B's interaction with parameter A positively impacted the output. It means the impact of the positive effect of throat diameter on response is more than the positive effect of contraction ratio on response.

**Fig. 8:**
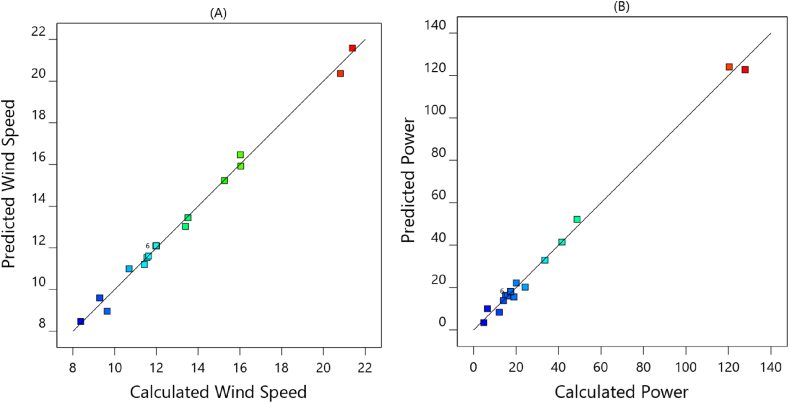
Comparison of calculated and predicted values of the designed model for (A) wind speed and (B) power.

The presented models fitted to the range of design parameters are given the mean of wind velocity and power in the duct throat with high accuracy. A correlation coefficient 0.8 has been introduced as a reliable boundary [33] to fit the data to the designed model. The high value of R^2^ denotes a significant convergence of the computed and actual data (Fig. 8). The determined coefficient (R^2^) for the regression equation in this study was more than 0.99 for two modeled response factors. An excellent connection between the parameters, their interactions, and their response is explained by the recently presented function. In Fig. 8(A) and (B), the projected and calculated values for wind power and velocity are shown for comparison. It can be seen from this figure; data are located along a straight line, which signifies that the computed and forecasted values for the two responses are similar. Consequently, these statements indicated that created models were appropriate for predicting the CFD results. They can be utilized to analyze and predict the wind speed and available power in ducted wind turbines.

**Fig. 9:**
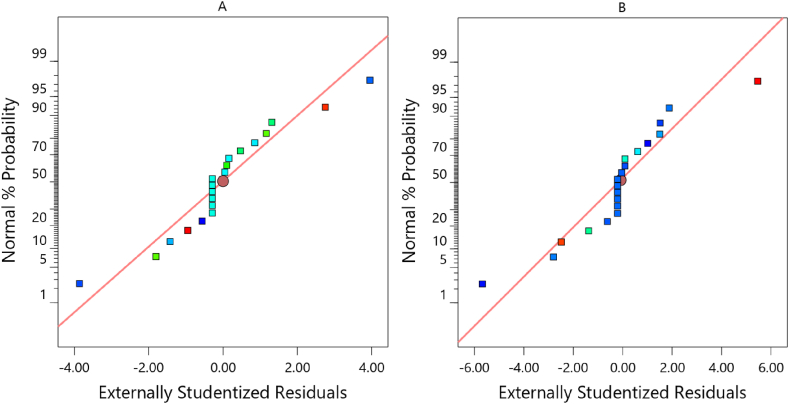
Chart of residuals with normal probability for wind speed (A) and power (B).

The standard probability plan of the studentized residuals is displayed in Fig. 9(A) and (B) as another technique to explore the impact of the specified model on wind velocity and power. The designed model is adequate if the standard probability plot points against the residuals are straight. Furthermore, the studentized residuals' standard distribution probability graphs were used as a useful tool to assess the suitability of the completed model. Fig. 9 demonstrates that the points follow a straight line and confirm errors get distributed appropriately. As all plots showed a relative constant variance in response ranges, the reported results showed no cause to dispute any independence violation or hypothesis of permanent variance.

**Fig. 10:**
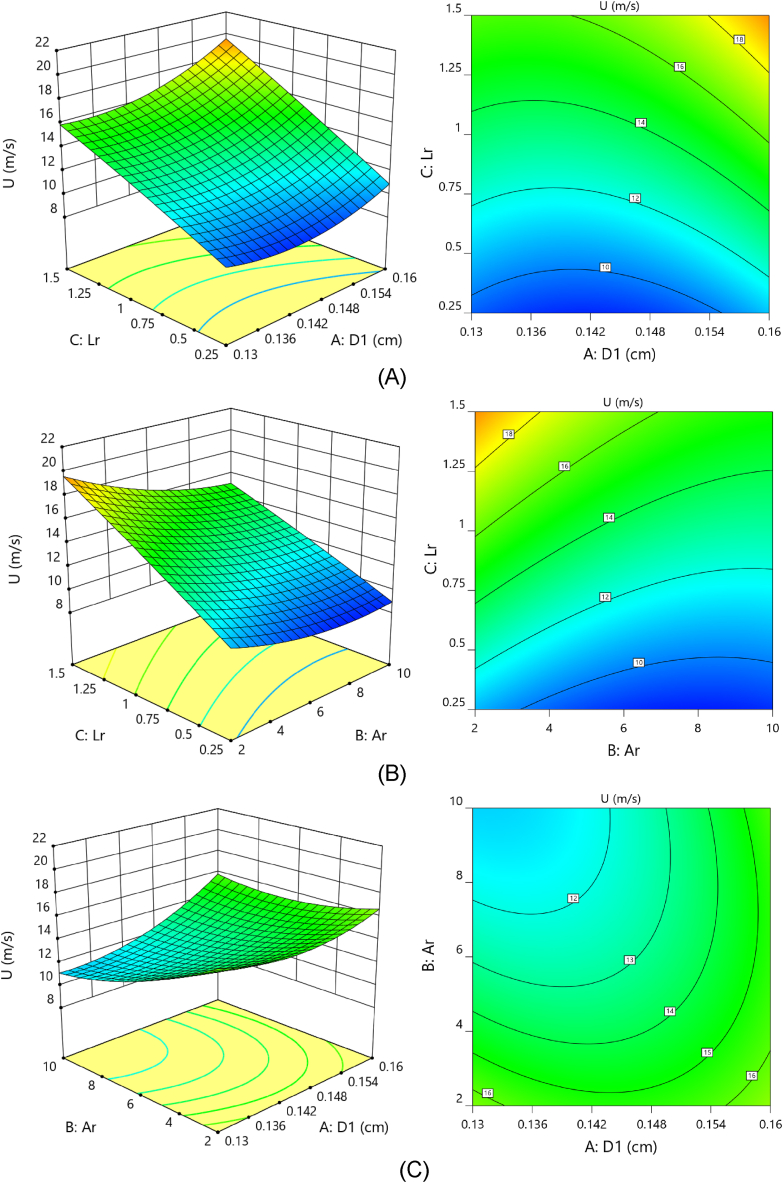
Improved wind speed 3-D response plots and contour analysis as a consequence of (A) duct neck diameter and duct length ratio, (B) expansion ratio and length ratio, and (C) expansion ratio and duct neck diameter.

Three-dimensional (3-D) surfaces can be used to examine how factors interact with response. To create 3-D surfaces, the Design Expert V.12 program was used. The 3-D surfaces were created to investigate the relationship among the model's variables (i.e., duct throat diameter, Contraction ratio, and length ratio). It can investigate the best range of each parameter to maximize the response in wind velocity and power. The varying labels on the contour plot and colors of the surfaces revealed the influence of changes in interaction related to wind speed and power levels. The reciprocal impact of the two factors on wind speed was greater than that depicted in Fig. 10. The length ratio and throat section diameter interact in the same way that the length and contraction ratios do. The curved slope of the surface indicates that the length ratio has a greater influence on wind speed than the duct throat diameter and contraction ratio. Then, this parameter has a vital function in boosting the wind velocity average measured in the throat section. It shows a matched result from the ANOVA's regression model. In other words, the flow stream requires enough length to develop and increase before entering the throat section. In this study, the increase in the length ratio is the main reason for the increasing wind speed inflow stream passes through the duct in the throat section. A rise in the length ratio with growth in duct throat diameter leads to a maximum value in enhanced wind speed (Fig. 10 (A)). As illustrated in Fig. 10 (B), any reduction in contraction ratio enhanced the wind velocity with a lower curve slope. With increasing the contraction ratio, a significant reduction in the enhanced wind speed occurs. This drop can be defined as a barrier in the wind flow, then, the wind speed rose as the contraction ratio decreased.

Fig. 10 (C) depicts the mutual effects of contraction ratio and duct neck diameter on increased wind speed. These two parameters have a more negligible effect on wind speed than two other interactions. Still, the effect of any change in each parameter is the same as reported in the previous analysis. The maximum wind speed is achievable at maximum throat diameter and lower contraction ratio.

**Fig. 11:**
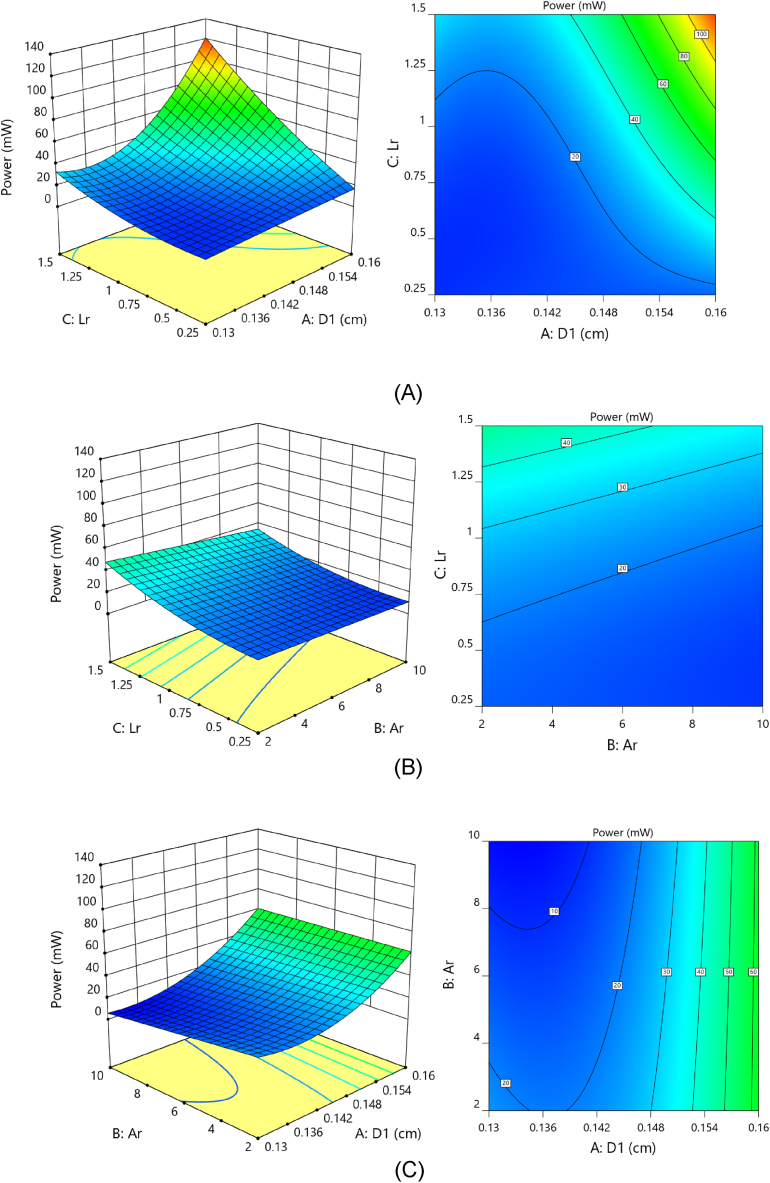
Available power response surfaces plots and contour analysis as a consequence of (A) duct neck diameter and duct length ratio, (B) expansion ratio and length ratio, and (C) expansion ratio and duct neck diameter.

According to the response surfaces illustrated in Fig. 11 (A), there is a strong correlation between duct neck diameter and length ratio. The power reaches its optimum level when the duct neck diameter and length ratio are both increased. Any decline in any of these will lead to a major loss of performance in power. As shown in Fig. 11(B) and (C), a contraction ratio with a low slope influences power, and as the ratio increases, the available power drops. On the contrary, when the length ratio and duct neck diameter increase, the output power level increases with a stronger effect. The limited interaction between the length proportion and the reduction in the ratio can be seen in Fig. 11. On the other hand, the relation between the proportion of length and the diameter of the duct's neck on output power is quite strong.

### 3.3 Optimal operating parameters for maximum power

The best performance parameters to increase power were investigated. One of several targets of this research was to check the effect of combining independent factors (L_R_, A_r,_ and D_t_) to achieve the maximum available power. Different combinations of design parameters were investigated by performing different optimization simulations using RSM to achieve the maximum available wind power. Based on the optimization, the maximum power available for the designed models was 124 W. It was obtained in the following conditions: the diameter of the duct's neck was 0.16 m, the index of concentration was two, and the index of length was 1.5.

We can pick the appropriate values for the needed operation from a set of input and response variables using mathematical optimization. The software provides various modes to define the optimal condition of the parameters. It includes lowest, highest, ranging, none, goal, and setting to establish the best outcome. The source parameters are determined in ranged data, and output (power) was set to get the maximum value in this study. Then the maximum available power and wind speed are achieved by a duct with a throat diameter, length ratio, and concentration ratio of 0.16, 1.5, and 2, respectively, as shown in Table 4.

**Table 4:** The best ranges for the response and specified parameters.

Variable	Target	Min	Max	Optimized value
Throat Diameter (m)	In range	0.13	0.16	0.16
Length Ratio	In range	0.25	1.5	1.5
Concentration Ratio	In range	2	10	2
Wind Speed (m/s)	Maximum	8.4	21.4	21.6
Power (W)	Maximum	4.8	127.8	124.1
Desirability	–	0	1	0.977

Compared to similar optimization, Allaei and Andreopoulos [47] reported that their duct can enhance the wind speed from 7 m/s to 12 m/s in the throat section (ratio of 1.71). In contrast, increasing wind speed to about 2.16 times free stream flow in the presented duct is possible.

**Fig. 12:**
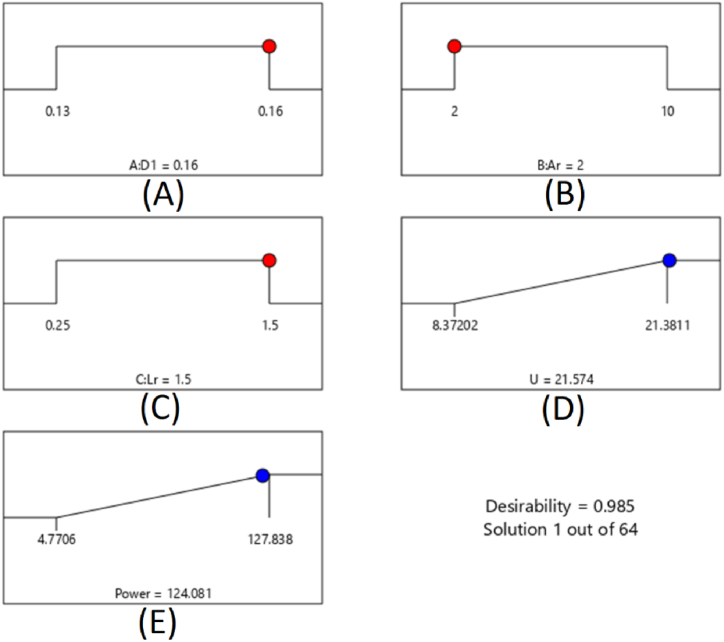
Desirability ramp for adjustment of settings for maximum available power, optimized values for (A) the Diameter of the duct's neck, (B) the index of Contraction, (C) Length ratio, (D) Wind velocity, and (E) available power in the duct's neck.

Fig. 12 shows that the created model's output values were near the model's maximum estimate. The acceptability rating ranges from 0 to 1, indicating how well the model output matches the desirable results. Then it explains how the specified variables are placed in the best way to guarantee the responses. The optimization is successful since the desirability value is close to 1 (0.977).

**Fig. 13:**
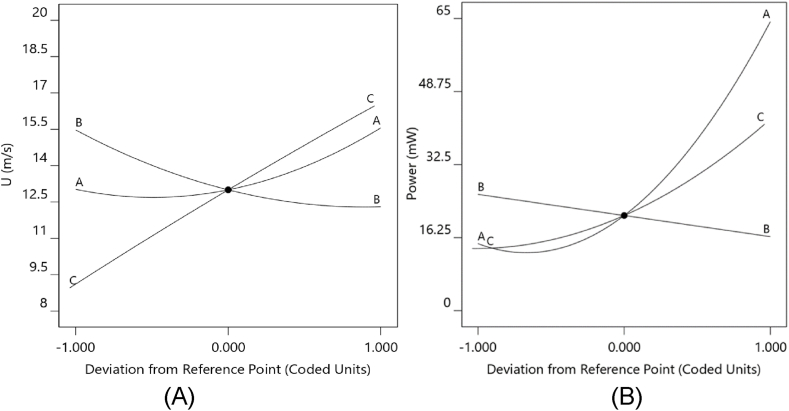
Deviation from reference points with coded factors.

To evaluate the impact of design variables at an optimized point, the perturbation plot was used [33]. The perturbation plot depicts the response surface's total influence on all design variables. In contrast, the center point (0) represents each parameter's middle of the performance range. Fig. 13 A depicts the perturbation plots used to test the influence of design variables (throat diameter, contraction ratio, and length ratio) at points that serve as references. Fig. 13 A shows that the rate of wind speed rise reduces as the contraction ratio increases due to the increment of the blockage effect against wind flow. It becomes obvious that the wind speed increases as any rise in throat diameter and length ratio occurs. Then it is possible to balance the blockage effect and provide enough space to develop the flow stream. However, the increment rate of wind speed was further for length ratio compared to the throat diameter. Fig. 13 B shows that the power increased with the throat diameter and length ratio. The impact of the length ratio is greater than that of the throat diameter. In comparison, the effect of the contraction ratio is on the opposite side of the two other parameters. It can be found from this plot; the wind speed and power are similarly affected by design parameters, while the rate of this effect is not the same for the two responses.

## 4 Conclusion

A numerical simulation created a novel model for high-performance duct-mounted wind turbines. The influence of three primary design variables, like throat diameter, concentration ratio, and length ratio, investigated the enhanced wind speed and the available power. The wind capture process was optimized to maximize the wind speed and available power in the installation location of the wind turbine using the CFD-RSM simulation−optimization method. The performance of the designed ducts was analyzed using CFD, and design parameters and their related responses were obtained using numerical simulations. The optimized condition for design parameters is considered to maximize the wind speed and available power in the duct throat using simulation−optimization RSM. The result was as follows:

1. The regression analysis of the design variables clearly shows that the models successfully fitted to calculated data. It specified the optimum value of variables to maximize available power and wind velocity at the duct throat.
2. The model p-value for wind speed and available power was less than 0.05, showing that the two designed models are significant. On the other hand, the model F-value for the wind speed and the power performances were 175.96 and 203.59, respectively. It signifies less than a 0.01% chance that noise can cause a significant amount of F-value to occur.
3. Optimizing the designed duct performance was done. The calculated data from CFD models for wind speed and power agreed with the developed model. The best available power values were obtained at throat diameter, contraction ratio, and length of 0.16 m, 2, and 0.16, respectively. Wind speed and power yields were 21.6 m/s and 124.1 W in optimized conditions.
4. RSM is a helpful strategy for optimizing ducted wind turbine-generated power to attain higher performance targets. RSM with strong predictive performance helps determine the appropriate performance of a duct for shrouded wind turbines. As a result, the difficulties of recurring practical trials and their associated expenditures are avoided. Quick and easy decision-making for developing the created ducted wind turbine applications is achieved. Comparing RSM predictions with CFD data shows that RSM can predict the performance and efficiency of duct-mounted wind turbines in a wide range of design parameters. However, the time and expense of CFD computations will be much reduced by prediction with RSM.

## Author contribution statement

Javad Taghinezhad: Conceived and designed the experiments; Contributed reagents, materials, analysis tools or data; Performed the experiments; Wrote the paper.

Shiva Abdoli, Walter Silva: Analyzed and interpreted the data; Wrote the paper.

Samira Sheidaei: Performed the experiments; Analyzed and interpreted the data.

Reza Alimardani, Esmail Mahmoodi: Conceived and designed the experiments; Wrote the paper.

## Data availability statement

Data will be made available on request.

## Declaration of competing interest

The authors declare that they have no known competing financial interests or personal relationships that could have appeared to influence the work reported in this paper.
